# RETRACTION: Causal Relationship Between Diet, Lipids, Immune Cells, and Chronic Fatigue Syndrome: A Two‐Mediation Mendelian Randomization Study

**DOI:** 10.1002/fsn3.71097

**Published:** 2025-10-17

## Abstract

**RETRACTION**: LiJ. , QinQ. , ZhuY. , QianY. , YinJ. , GaoX. , WenH. and WangP. , “Causal Relationship Between Diet, Lipids, Immune Cells, and Chronic Fatigue Syndrome: A Two‐Mediation Mendelian Randomization Study,” Food Science & Nutrition 13, no. 6 (2025): e70424, 10.1002/fsn3.70424.40552329 PMC12183343

The above article, published online on 22 June 2025 in Wiley Online Library (wileyonlinelibrary.com), has been retracted by agreement between the journal Editor‐in‐Chief, Y. Martin Lo; and Wiley Periodicals, LLC. The retraction has been agreed upon following an investigation into concerns raised by a third party. The investigation concluded that the article does not sufficiently present the biological rationale required to justify the use of Mendelian Randomization for the research question, as outlined in the STROBE‐MR guidelines cited by the authors. Furthermore, the article contains inconsistencies in how cheese consumption is described and its reported association with chronic fatigue syndrome, including distinctions not supported by the methods or results. Additionally, the article's abstract claims an association between “breakfast affinity” and chronic fatigue syndrome, but this is not supported elsewhere in the text or figures. Finally, the article lacks details on any correction for multiple testing, despite the large number of variables examined. The authors cooperated with our investigation, explaining that these errors and omissions were inadvertent. They also provided additional data; however, this was not deemed sufficient to restore confidence in the article. As a result, the editor considers its findings unreliable. The authors disagree with the retraction.

**RETRACTION**: J. Li , Q. Qin , Y. Zhu , Y. Qian , J. Yin , X. Gao , H. Wen and P. Wang , “Causal Relationship Between Diet, Lipids, Immune Cells, and Chronic Fatigue Syndrome: A Two‐Mediation Mendelian Randomization Study,” Food Science & Nutrition 13, no. 6 (2025): e70424, 10.1002/fsn3.70424.40552329 PMC12183343

The above article, published online on 22 June 2025 in Wiley Online Library (wileyonlinelibrary.com), has been retracted by agreement between the journal Editor‐in‐Chief, Y. Martin Lo; and Wiley Periodicals, LLC. The retraction has been agreed upon following an investigation into concerns raised by a third party. The investigation concluded that the article does not sufficiently present the biological rationale required to justify the use of Mendelian Randomization for the research question, as outlined in the STROBE‐MR guidelines cited by the authors. Furthermore, the article contains inconsistencies in how cheese consumption is described and its reported association with chronic fatigue syndrome, including distinctions not supported by the methods or results. Additionally, the article's abstract claims an association between “breakfast affinity” and chronic fatigue syndrome, but this is not supported elsewhere in the text or figures. Finally, the article lacks details on any correction for multiple testing, despite the large number of variables examined. The authors cooperated with our investigation, explaining that these errors and omissions were inadvertent. They also provided additional data; however, this was not deemed sufficient to restore confidence in the article. As a result, the editor considers its findings unreliable. The authors disagree with the retraction.

